# A Multiagent System for Dynamic Data Aggregation in Medical Research

**DOI:** 10.1155/2016/9027457

**Published:** 2016-11-16

**Authors:** Alevtina Dubovitskaya, Visara Urovi, Imanol Barba, Karl Aberer, Michael Ignaz Schumacher

**Affiliations:** ^1^Applied Intelligent Systems Laboratory, HES-SO VS, Sierre, Switzerland; ^2^Distributed Information Systems Laboratory, EPFL, Lausanne, Switzerland; ^3^Accounting and Information Management, Maastricht University, Maastricht, Netherlands; ^4^Information Security Group, UPC, Barcelona, Spain

## Abstract

The collection of medical data for research purposes is a challenging and long-lasting process. In an effort to accelerate and facilitate this process we propose a new framework for dynamic aggregation of medical data from distributed sources. We use agent-based coordination between medical and research institutions. Our system employs principles of peer-to-peer network organization and coordination models to search over already constructed distributed databases and to identify the potential contributors when a new database has to be built. Our framework takes into account both the requirements of a research study and current data availability. This leads to better definition of database characteristics such as schema, content, and privacy parameters. We show that this approach enables a more efficient way to collect data for medical research.

## 1. Introduction

Research studies that use retrospective medical data have become a major source of contributions to the biomedical science literature [1]. Clinical data repositories are promising resources for the development of personalized medicine, clinical trials, epidemiology, and public health [2]. Unfortunately, the collection of medical data is notoriously time-consuming. Data collection in one medical institution may take several years [3]. In order to accelerate this process, or when required data are diverse and cannot be collected on site, multiple medical institutions may collaborate to aggregate the data. However, distributed medical data aggregation is challenging as it requires solving privacy and data quality issues, as well as enabling interoperability between medical systems.

According to the data protection legislation in Europe and US, collecting and sharing personal data require signed consent from the patient to allow using data for research purposes [4, 5]. Not all patients are willing to provide a consent because of the sensitive nature of their medical data. For example, if the data become publicly available insurance companies may infer that a person is suffering from a chronic disease and may refuse an application or reject the renewal of their insurance policy. An employer may try to infer healthcare information about potential employees and based on the sensitive information (a serious health condition or a chronic disease susceptibility) may discriminate the candidate.

As an alternative to the consent collection, the data can be anonymized to be used in clinical research [4, 5]. This could be done by applying existing privacy protection mechanisms [6–9]. However, mobility of the patients and a will or sometimes a necessity to visit more than one medical institution can introduce another privacy threat. It has been shown that in the case of the independent release of locally anonymized datasets that contain information about the same patients their reidentification is still possible (e.g., in the case of a composition attack first described in [10]). In order to counter these privacy threats, several models in the area of distributed privacy-preserving data publishing have already been proposed (i.e., pseudonymization [6, 7], secure multiparty computations [8], microaggregation [9], and cloning [10]). However, those models can significantly affect the quality and, therefore, the utility of data, since they do not take into account data availability, content, structure, and representation.

Both the structure and the representation of the health data that need to be aggregated for the research purposes depend on the requirements of a study. Therefore, it is not possible to specify a unique static schema of the database that will fit different clinical studies. In order to guarantee the data utility and patients' privacy, the database schema and privacy parameters have to be adjusted based on the clinical study, for which the database will be employed.

Building multiple databases for different research studies is, for example, particularly relevant to one of the key concepts of personalized medicine: therapeutic drug monitoring (TDM) [11]. TDM transformed drug therapy by providing the ability to characterize sources of variability in drug disposition and response to individualize drug dosing [12]. TDM is based on models that allow the computation of the characteristics of a particular drug based on the patient's covariates. In order to build these models, population healthcare data are needed. The data requirements vary for different drugs and populations (e.g., neonates or adults), and therefore multiple databases need to be constructed.

We aim to develop a system that will connect researchers and medical institutions and will allow them to collaborate with each other. This paper presents a multiagent system (MAS) for dynamic data aggregation in medical research. We use agents as the problem requires a distributed and autonomous system, where participants can join the network and decide what to search for and what to share independently from the other participants of the network. The participants do not necessarily know each other and may use different ways to structure their data. By representing participants as autonomous agents in a distributed network, we can then focus on defining all the mechanisms for coordinating the participants to find each other and to share the data in a meaningful way. The system (i) enables the connection of research and medical institutions into a peer-to-peer (P2P) network and (ii) provides an environment to negotiate and define the characteristics of the database such as schema, content, and privacy parameters based on the data requirements and availability.

We evaluate our system using patients data collected in the neonatal intensive care unit over 5 years within the frame of a routine TDM program [3]. The advantages of our solution are the following:

- A research study can be conducted faster, as the time needed to aggregate the required amount of data is dramatically decreased in the case of using our system with respect to the time needed for data collection in a medical center.
- Multiple databases (satisfying the requirements of different research studies) can be shared between the users of the proposed system: medical and research institutions.
- The sensitive nature of medical data is considered during every step of data aggregation in order to achieve trade-off between privacy and utility.
- The system is “fair” in the following sense: if all users participate in data aggregation, every user will be able to gain access to approximately the same amount of data as he/she contributes. It means that every user of the system can benefit from the data collection. We believe that this will motivate medical and research institutions to join the system and participate in data aggregation.

The rest of the paper is organized as follows. In Section 2.1, we provide a use-case scenario and a general description of our framework. In Section 2.2 we demonstrate* dynamicity* of our system: we present in detail the process of P2P network organization and the agents' negotiation phase. We also provide the necessary background about existing coordination models we build our negotiation mechanism on. In Section 2.3, we discuss privacy and security concerns. We provide the description of the implementation and evaluation results of the system in Section 3. In Section 4, we compare our approach with the related work. We conclude and list the directions of our future research in Section 5.

## 2. Materials and Methods

### 2.1. MAS Framework

In this section we show how our system could be used by medical and research institutions. We also present the architecture of the system and describe functionalities of its elements.

#### 2.1.1. Use-Case Scenario

There is growing interest and a strong need to share individual patient data for secondary purposes, particularly for research [13]. The system presented in this paper will facilitate and accelerate the data sharing and aggregation. We assume the following scenario. Users of the system are research institutions and medical doctors or healthcare institutions that possess the medical data. Users may have the following goals: (1) to access anonymized medical data and use them in particular research study and (2) to contribute to the development of research by sharing patient data. For simplicity we assume that there is no economical competition between different research and medical institution.

#### 2.1.2. MAS Architecture

**Figure 1:**
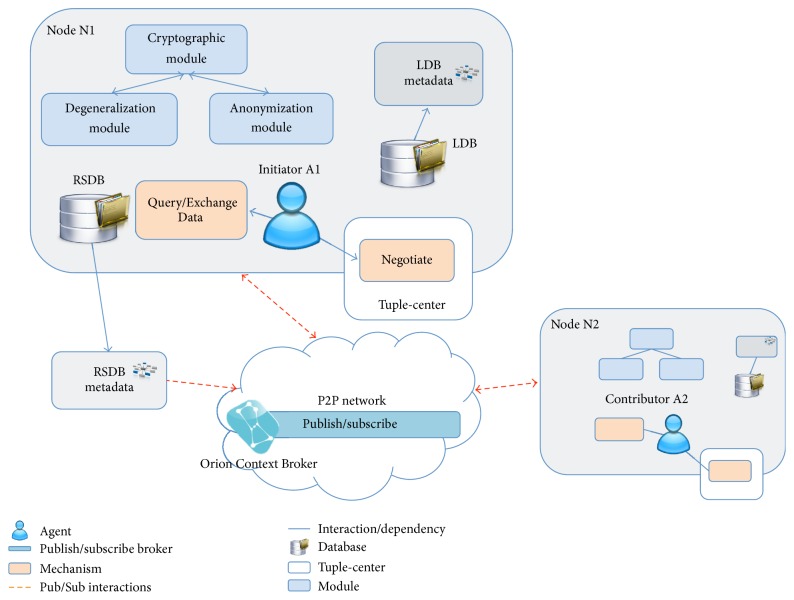
Architecture of the multiagent system.

Figure 1 presents an architecture of our multiagent system for dynamic data aggregation and its components and their relationships with each other and with the environment. It consists of a publish/subscribe broker that serves as a lookup system and the nodes that represent users of the system. Based on the user's requirements, one or several* agents* could be initialized by the node.* Agents* are used at different stages of the process of building a research database (RSDB) from distributed local sources (LDB), first, to find the contributors to the database and, second, to adjust the structure and representation of the data depending on the requirements of a particular research question, current availability of the data, and privacy considerations. These steps require coordinating the participants, interactions, and reasoning; therefore, we employ agent-based approach.

An RSDB is a database with anonymized data to be used for research purposes. Each RSDB will be constructed taking into account the requirements of a particular research study; for example, in case of TDM this could be the concentration measurements of a specific drug in the patient's blood. The information about already constructed RSDB (metadata of RSDB) will be shared within the network; therefore, there is no need to aggregate the data again if a similar research study has to be conducted. A user will be notified if there exists a database that satisfies the user's requirements.

LDB contains patients' data collected in a medical center. This information will only be aggregated after coordination, agreement on the characteristics of the database, and applying privacy and security mechanisms. Metadata of LDB consist of the information that describes medical data stored in LDB and used to identify the potential sources for an RSDB. No sensitive information can be shared during organization of P2P network and agents coordination.

The nodes can interact with publish/subscribe broker to either publish the availability of the data or make a subscription based on the requirements of a research study. If a new database has to be constructed we need to identify the sources of the data and to connect them. For this we use the publish/subscribe paradigm to discover the nodes with relevant data instead of multicasting a request. More information about the process of P2P network organization is provided in the next section. Nodes can have access to their LDBs and can use the functionality of the following mechanisms:* Query/Exchange Data* and* Negotiate. Query/Exchange Data* is used to publish and subscribe using the broker, to query and exchange the metadata, and to transfer the data to an RSDB.* Negotiation mechanism* is based on the TuCSoN coordination model [14] and aims at adjusting the characteristics of RSDB (e.g., the schema of the database, required number of records to be collected, and privacy parameters). We will focus on coordination between agents in the next section. As a part of the negotiation process a semantic agreement between schema of different databases and different data representations needs to be established. This is out of the scope of our work; we assume that existing ontologies and schema matching solutions [15, 16] can be employed.

To ensure authenticity, integrity, and anonymity of the data that are being aggregated, we developed the following modules: cryptographic module, anonymization module, and degeneralization module. The functionalities of the cryptographic module are (i) to create pseudonyms with which the data about the patient will be uploaded to RSDB and (ii) to generate the signature before data transfer in order to ensure the authenticity and integrity of the data. The anonymization module uses generalization algorithms that allow replacing the exact values of the data with a range within which these data fall. This guarantees* k*-anonymity property in a distributed environment and therefore ensures the data privacy. The algorithms are described in detail in [17]. Degeneralization module will be implemented to improve the quality of the data with the growth of RSDB by mitigating the data losses due to applying anonymization algorithms.

The dynamics of a system is characterized by constant change, activity, or progress (http://www.oxforddictionaries.com/definition/english/ dynamic). The term* dynamicity* in the context of complex open and distributed systems can be intuitively defined as the ability for a system to be configured, developed, maintained, and modified at runtime, without compromising its integrity and ongoing processes [18]. We use the term dynamicity in the following sense. First, we assume that the number of agents participating in the data aggregation is not static: that is, an agent may join and leave the network. Second, we use term dynamicity to specify that there is no need to have fixed static description of the data to be aggregated. It can be adjusted during negotiation phase. Dynamicity allows one to accelerate data collection process. Hereafter, we describe two main interaction processes: a publish/subscribe mechanism, which helps agents to get organized in a P2P network (Section 2.2.1), and negotiation: a process that allows agents to find an agreement on the data representation as well as security and privacy parameters (Section 2.2.2).

### 2.2. Dynamicity of MAS

#### 2.2.1. P2P Network Organization

We use a publish/subscribe paradigm to organize the nodes in the P2P network. It allows delivery of the data from their producers (publishers) to their consumers (subscribers) in the distributed environment in a decoupled fashion [19]. This means that publishers can introduce the data into the system (publish/subscribe broker) being unaware of the subscribers. Subscribers can register their interests by subscriptions, which filter relevant events to the subscribers. The broker enables publication of context information by publishers, so that the relevant information becomes available to subscribers.

**Figure 2:**
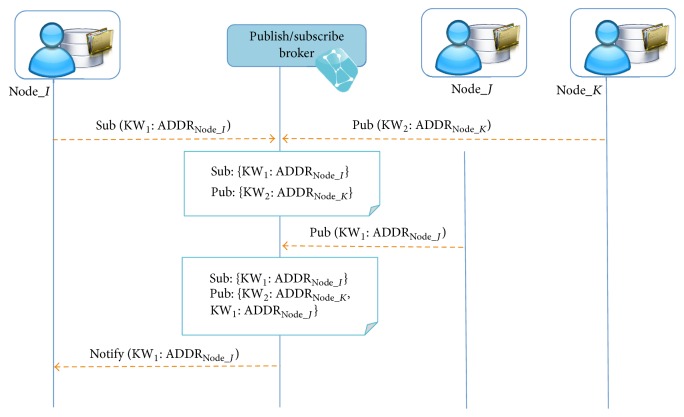
Process of peer-to-peer network organization.

The role of a publish/subscribe broker in our system is to support dynamicity and to allow the node (i) to register availability of a certain kind of medical information within the network and (ii) to subscribe for a notification if a certain type of information has been published. This is done to avoid performing active discovery of peers or forcing the publishing nodes to broadcast the network to demonstrate data availability each time when there are new peers joining the network.  Figure 2 illustrates the P2P network organization mechanism used in our framework. It shows how we structure the messages that are used during the interactions between the broker and the nodes.

After registering at the broker the node subscribes to a certain type of data by specifying a set of keywords (KW = [kw_1_,…, kw_*n*_]) that describe the data the node is interested in. Similarly, for a node that possesses the data, it is sufficient to publish the description of the data using the keywords. If the keywords match corresponding subscriber will be notified by the broker and provided with the list of the addresses of the nodes with relevant data available. The semantic description of the data has to be provided by the users of the system. This is why we have chosen a simple keyword approach. In the future work we plan to improve the mechanism for P2P network organization.

#### 2.2.2. Agents Negotiation

Negotiation is a process initiated by a node in order to obtain a certain number of medical records to build an RSDB for a particular research study. It is followed by the process of discovery of the nodes with the relevant data. Negotiation is built on interactions between agents within the TuCSoN coordination model [14] that is happening through tuple-centers (TCs). TCs can be seen as a shared system such as blackboard system [20] where the information is being exchanged in form of tuples. The templates of the tuples need to be specified with respect to their structure. An ontology model could also be employed to interpret the information transferred by the tuples. We will describe the structures of the tuples at different states of the negotiation below.

**Figure 3:**
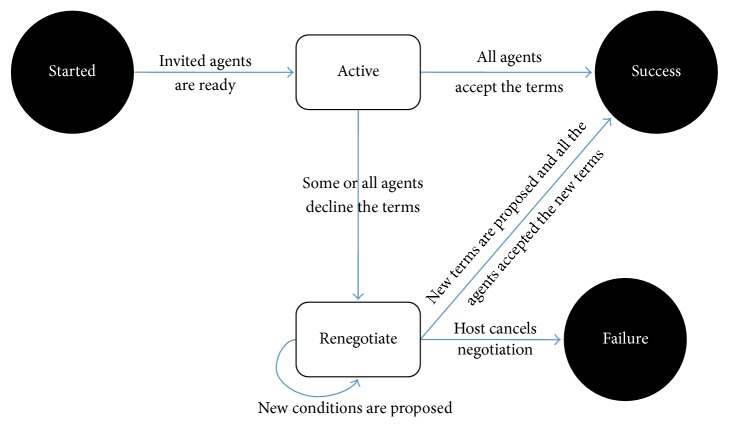
States of the negotiation process.

Using the tuple-center, an agent can, for instance, write (out operation), read (rd operation), or consume (in operation) the tuples.  Figure 3 presents a state diagram for the negotiation process proposed in the paper and implemented as a part of our framework. It demonstrates the states and the transitions between them.

The node that initiates the process of data collection creates a Negotiating Agent (host) to start the process of negotiation. Next, the host creates a TC within its own node, where the negotiation will take place. Once the TC has been successfully created, the agent injects a script that controls the state of the negotiation. The script is written using the first-order logic language ReSpecT [21] and allows programing the behavior of a TC. The following states are possible during the negotiation process:

(i) *Started*. The host writes into the TC a tuple with respect to the predefined template that consists of a list of agents ($t_{1}=invited$(*AgentList*)) that will be invited to take part in the negotiation. When an agent from the list arrives to the TC, it writes a tuple $t_{2}=hello$(*AgentId*).
(ii) *Active*. When all the agents write the tuple *t*_2_, a reaction that sets the state to* Active* is triggered. At this stage, the node proposes the conditions of the negotiation and the peers evaluate them using the rules. In the case of building the database, the conditions could be the schema of the database (attributes and their ranges), number of records needed (*N*), and privacy parameters if required. A node writes the following tuple specifying *m* attributes (attr), number of records *N*, and keywords:
  (1) t3=parametersKW,attr1,min1,max1,…,attrm,minm,maxm,N.
 Then, the conditions have to be evaluated by the other nodes using rules; for example, the agent *J* reads the tuple from the agent *I* and evaluates it as follows:
  (2) KWI⊂KWJ∧attrpI=attrpJ∧minpJ≤minpI∧maxpJ≥maxpI,  p∈1,m∧NJ≥0.
 If the conditions are satisfied then the agent will write the following tuple:
  (3) $$\begin{array}{c} t_{4}=answer\left(\;\text{AgentId},\tilde{N}\;\right), \end{array}$$
 where $\tilde{N}$ is a number of records an agent (with corresponding* AgentId*) can contribute to the RSDB. We provide an example of the tuples and conditions that we used during evaluation in Section 3. A threshold for the peers to respond is used to bound the maximum duration of this state.
(iii) *Renegotiate/Failure*. If the conditions of data exchange proposed by the host are not accepted by one or more peers, it is possible to either terminate the negotiation by setting it into the* Failure* state and marking the TC as reusable or set the state to* Renegotiate*. At this state the list of participants could be changed, and the peers can modify the parameters of the tuples. Currently, acceptance of the terms is based on the user engagement. When the* Failure* state is reached, all agents terminate.
(iv) *Success*. If the terms are accepted by all the peer agents, the data transfer occurs. When each node finishes data transfer to the host, the host marks the agent as finished writing a tuple $t_{5}=finishedAgent$(*AgentId*). When all the agents from the invite list have been marked as finished,* Success* state is triggered, effectively ending the negotiation process as all agents terminate when this state is reached.

In the end of this process, either the host agent will obtain a sufficient amount of data or it will be waiting for other (or existing) peers to join the negotiation again to complete aggregation of data. The host will be notified by the broker if an agent publishes at the publish/subscribe broker information about the availability of the data. Then the agent will be able to join the negotiation process. The state will return to* Active*, repeating this cycle until the host obtains the desired amount of data.

For the sake of simplicity we do not present the structure of all the tuples that we use to model the reactions in the cases such as removing an agent from an active negotiation process or changing the status of the negotiation process.

### 2.3. Data Security and Privacy

Hereafter we discuss privacy and security requirements to the medical data before they could be transferred in the case of distributed data aggregation for the research purposes. We also describe how we are going to address the need for privacy and utility trade-off in our system.

#### 2.3.1. Need for Security and Privacy

In order to be sure that the research database contains only veritable medical data, it is very important to provide integrity and authenticity of the data, that is, to insure that the data are correct, the data source is a real medical institution, and it is possible to recontact the doctor that provided the data (if needed). Therefore, the certification authority needs to be deployed and every time the data are sent to the research database the use of digital signature [22] is required. These methods are standardized, and their functionality can be provided through the cryptographic module at every node.

As already mentioned it is impossible to have one fixed data structure for different types of medical research. Therefore, privacy-preserving mechanisms need to be adapted for different datasets. In [23] authors proposed the notion of* k*-anonymity: ensuring privacy by constructing a set of *k* records indistinguishable in terms of QID quasi-identifiers, a set of the attributes that can (in combination) identify a person. This approach is based on applying generalization functions to QID and suppression to uniquely identifiable patients data. *k*-anonymity guarantees that the probability to deidentify a person to whom a record belongs does not exceed 1/*k*, where *k* is the cardinality of the set of indistinguishable records.

#### 2.3.2. Privacy-Utility Trade-Off

Anonymization certainly affects the data utility [24]; therefore it is of high importance to be able to adapt privacy parameters taking into account the format of the data that will be collected. The utility expectations should be specified depending on the requirements of a particular research question. And this will be base for defining privacy parameters and the generalization functions for each of the attributes from QID.

In our MAS the values of the privacy parameters can be seen as one of the conditions specified by host based on the utility expectations. Every contributor can propose to modify the parameters during the process of agents negotiation described in Section 2.2.2. In [17] we proposed algorithms that allow the release of medical data for the research purposes from different LDBs independently, while preserving the anonymity property of RSDB. Generalization rules are expressed as binary trees and are used to achieve *k*-anonymity and maximum utility without revealing nonanonymized QID values to the system. We ensure that given the consent of the patient caregivers will be able to update RSDB with the data about the patient without creating multiple entries that correspond to the same person. Our solution also relies on pseudonyms and provides a possibility to recontact the patient through a caregiver that uploads the data. This functionality can be used by an agent that can now employ anonymization before making a contribution to the RSDB.

#### 2.3.3. Data Transfer

Before the data are transferred the anonymization algorithms [17] are applied. This guarantees that *k*-anonymity of RSDB is preserved, and, therefore, patient privacy will not be violated. The data are transferred using a separate web service. When a new RSDB is constructed, its metadata are sent to the broker and are kept updated. This allows one to reuse the database if needed or populate it with more records, keeping the data consistent and private.

## 3. Results and Discussion

In this section we provide the details about development and virtualization environment that has been built in order to implement the MAS described above. We describe the datasets that have been used to evaluate consistency, performance, and scalability of the system. The results of the evaluation are discussed in Section 3.4.

### 3.1. Development and Virtualization Environment

For the system development the Java language has been chosen based on the following reasons: the programming API of TuCSoN is written in Java, a high-level language is required to program the complex tasks the agents perform, and execution is reasonably fast. The machine used for development runs GNU/Linux, specifically, Xubuntu 14.04. The system runs on top of a VT-x capable Intel Xeon CPU with 8 logical cores and 16 GB of RAM.

**Figure 4:**
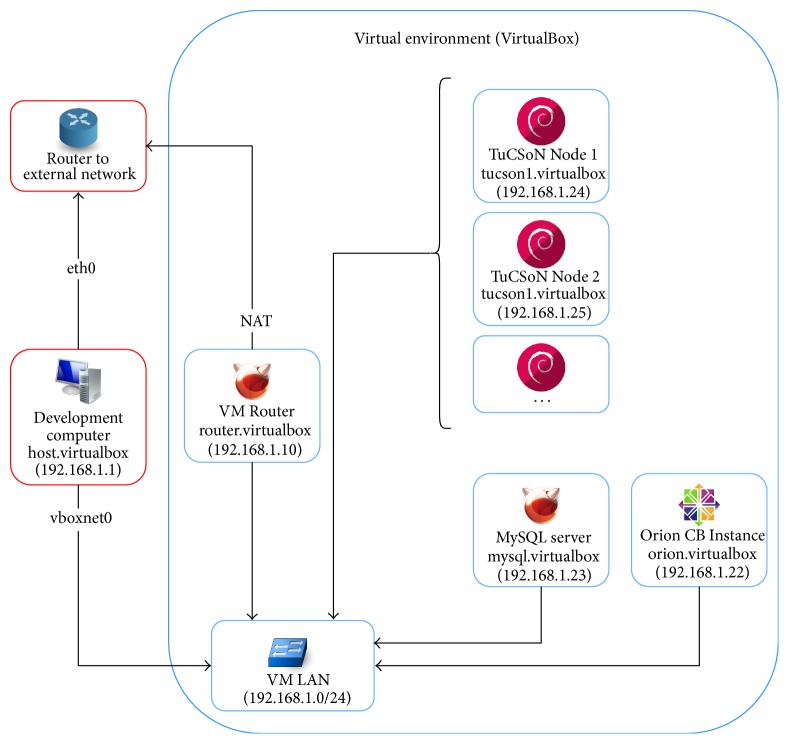
Virtualization environment.

To test the system, a virtualized environment was set up with VirtualBox 5.0.1 used as virtualization engine. As shown in Figure 4 the virtualization environment is comprised of several virtual machines and a host-only network, which isolated the virtual machines and the external network environment to avoid unsolicited traffic interfering with the virtualization environment. Outbound access from this network was routed through a virtual machine hosting a DHCP server and DNS server. All the virtual machines were running with a KVM-compliant paravirtualization layer and hardware-assisted virtualization through Intel VT-x. Virtual machines running FreeBSD as guest OS had a Hyper-V layer instead.  Table 1 illustrates the setup of the virtualization environment.

Table 1 shows the functionality and characteristics of each virtual machine used in the implementation. One has to notice that for the evaluation we deployed a single MySQL instance into which each node operates using its own database. In a real-life scenario, each node would have its own storage backend located at each node.

### 3.2. Dataset

The dataset is comprised of two separate databases, one with 8898 records (called* Gentamicin_large*) and a second one with the extended schema, containing more health information within 224 records (called* Gentamicin_small*), the database with 9122 medical entries in total. The data has been collected in preterm and term newborns treated with Gentamicin (an antibiotic) in the neonatal intensive care unit at the University Hospital Center of Lausanne and has been used both for the treatment and later on for the research purposes in the framework of the ISyPeM2 project (http://www.nano-tera.ch/projects/368.php). The data had been previously statically anonymized in the hospital as it is impossible to deidentify patients. The attributes of a record are the following: a pseudonym of the patient, body weight, gestational age, postnatal age, gender, and various information related to the concentration measurements of an antibiotic in the patient's blood. Based on the semantics of the data we annotated the dataset with the following keywords: “Gentamicin” and, “neonates”. The following attributes have been chosen: body weight (BW), gestational age (GA), postnatal age (PNA), gender, and concentration. We discarded some records that had missing values corresponding to any of the attributes listed above. This reduced the size of the resulting dataset to 8922 records.

To diversify subscriptions and the data that the nodes have we added some synthetic datasets annotated with the keywords “Malaria”, “adults”, “cancer” with the attributes age and gender.

### 3.3. Evaluation Scenario

To the best of our knowledge there is no system to benchmark with since existing systems do not provide the same functionality or work in different environments (see Section 4 for comparison with existing solutions). Therefore, we proposed the following evaluation scenario. We first test consistency, performance, and scalability of our system. Second, we would like to prove our initial assumption that the system is “fair” meaning that an agent that participates as a data provider can also obtain the data it needs. And the more the system is used the closer to the equality the amount of data an agent could provide and obtain.

We defined a set of 20 hardcoded conditions that differ from each other in values and combinations of body weight, gestational age, and gender. For example, ({“*Gentamicin”, “neonates*”}, {(*“BW”, 2000, 3000*), (*“GA”, 38, 42*), (*“gender”, any*), (*“concentration”, any*)}*, 6000*) expresses the conditions for the dataset containing 6000 records about neonates with bodyweight between 2000 g and 3000 g, gestational age from 38 to 42 weeks, and any gender and any concentration value.

To test consistency we would like to compare the results of using the same condition (selected randomly from the predefined set) in the case of querying the database directly (equally to 1 agent or to having data locally) and in the case when the data are distributed between 3, 5, and 10 agents. We make a realistic assumption that the number of participants for populating one database would rather not exceed 10; however, the number of data publishers is not limited by our system. We assume that there is always 1 agent that acts as a subscriber and all the other agents are publishers. The subscriber may also possess the data and make a contribution to the database. We evaluate performance and scalability by measuring the time of a system run, *t* ^run^(*n*), for different number of agents, *n*, that are ready to provide the data. We consider the running time as a time between the moment when subscriber in P2P network receives the notification about the data available and the moment of the dataset creation.

We evaluate “fairness” of our system by estimating “gain” and “loss” for every agent, participating in the data exchange for different number of agents. We simulate the settings in which every agent randomly selects a condition from the predefined set and initiates the process of dataset creation. We split the data randomly between 10 nodes: we populated each node's database with approximately 800 records. We then calculated an average difference between the number of records obtained and the number of records provided by a single agent while using our system after different number of runs. To avoid contingency we averaged out the results over all the nodes participating in the data exchange.

### 3.4. Evaluation Results

As expected for each condition, the numbers of records obtained from the databases from distributed sources, including the database of the initiator, always sum up to the number of cases obtained from the querying database before splitting the data. Therefore there is no data loss and the system is consistent.

The results obtained while evaluating scalability and performance are presented in Table 2. Table 2 shows that the system is scalable, and yet the time of the system run increases with the number of agents; it does not exceed one minute in the case of 10 agents. Important notice is that before aggregation is possible, the data have to be available locally: already collected by a medical center. Nevertheless, our system significantly decreases the amount of time needed to collect the required amount of data. Hereafter we compare the time of data collection performed entirely on site (in one medical institution) with the time needed to collect the same amount of data using our system that allows connecting *n* different medical institutions. We also discuss the results presented in Table 2.

The required amount of data that need to be collected for a specific research question can be expressed as a number of records, corresponding to different patients, or in case of TDM as a number of concentration measurements of a specific drug in the patient's blood. (If we consider different medical records we should take into account that the information about the same patient can be stored in multiple databases. To avoid multiple entries in the RSDB corresponding to the same patient cryptographic and anonymization modules have to be used.) Let us consider that we are interested in obtaining *D* measurements. We can assume for simplicity that each medical center or laboratory performs at least some certain number of tests per month, *r*. Then the time *t* ^loc^ needed to collect *D* measurements in one medical institution can be expressed as

(4) $$\begin{array}{c} t^{loc}=\frac{D}{r}. \end{array}$$

If we have access to multiple data sources (*n* local databases) then during one month there will be *n* × *r* tests available. Therefore, we can define the time needed to obtain *D* measurements from *n* databases, *t* ^dist^(*n*) (taking into account the time of a system run, *t* ^run^(*n*)). Then we can compare it with the time *t* ^loc^ needed to collect the same amount of measurements in one medical institution.

(5) tdistn=Dn×r+trunn,

(6) tloctdistn=Dr÷Dn×r+trunn.

Local data collection usually requires months, but as Table 2 shows the time of a system run, *t* ^run^(*n*), does not exceed a minute up to 10 agents (*n* = 10). This allows us to simplify (6), as *t* ^run^(*n*) is negligible compared to *t* ^loc^:

(7) $$\begin{array}{c} t^{dist}\left(\;n\;\right)\approx \frac{t^{loc}}{n}. \end{array}$$

Equation (7) shows that the time required for distributed data aggregation performed using our system, *t* ^dist^(*n*), is approximately *n* times less than the time *t* ^loc^, needed for on-site collection of the same amount of data, *D*. For example, for the dataset we used for the evaluation collection of the data in one medical institution took approximately five years [3]. Using 10 sources of data, for instance, would allow one to collect approximately the same amount of information we used for the evaluation during half a year instead of five.

**Figure 5:**
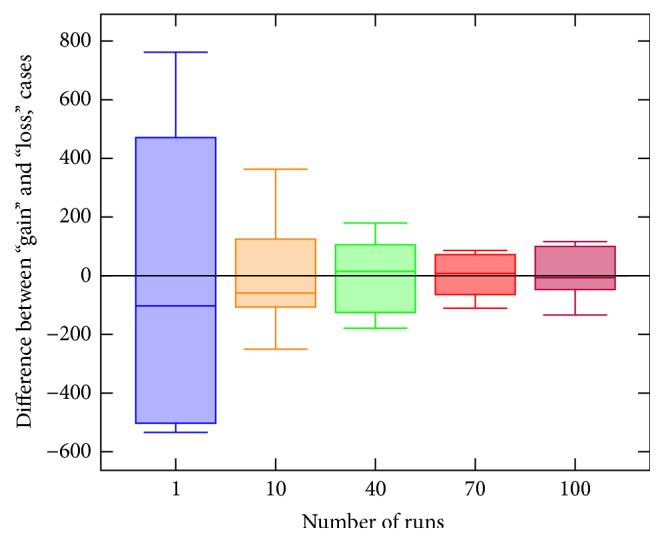
Simulations. The graph shows how the difference between the amounts of data provided and obtained by an agent changes with the increasing number of system runs.

To show the “fairness” of the system the results of the simulations with the 10 agents setup are shown in Figure 5. We measured the difference between “gain” and “loss” for every agent for the increasing number of runs. Negative values indicate that after a number of runs an agent provided more records than it obtained, while positive values show the opposite. We noticed that some nodes do obtain or do provide more cases than others, but on average the difference is low. Furthermore, we can see that the average difference between the number of records provided and the number of records obtained during the use of the system decreases with the increasing number of runs. Therefore, we have shown that the more time the system is in use the closer it is to a “fair” state, that is, when the difference between the number of records provided and the number of records obtained by an agent converges to zero.

## 4. Related Work

Comparative effectiveness research (CER) (https://www.nlm.nih.gov/hsrinfo/cer.html) is the conduct and synthesis of systematic research comparing different interventions and strategies to prevent, diagnose, treat, and monitor health conditions. In an effort to address a demand for an interinstitutional CER there have been new designs and implementations of informatics platforms that provide access to electronic clinical data. Sittig et al. [25] provide an overview of six platforms proposed as a result of collaborative work of different organizations such as hospital systems, pharmacies, healthcare players, and laboratory organizations.

Only one platform among six studied in [25] provides publicly available data. However, this data can only be used for the healthcare quality assessment. Another platform described by Sittig et al. was presented in the survey at its planning stage and we could not find any information available. Four other solutions provide the platforms for the research projects to be conducted in collaboration between selected medical centers on a study-by-study basis without support of dynamicity. In this case access to the data is granted only to the group of people involved in the particular project only, with an exception for the project i2b2 [26] where a deidentified training dataset can be accessed from the local network of the organization hosting the platform.

Elger et al. in their work [6] provide an overview of technical, practical, legal, and ethical aspects of secondary data use and discuss their implementation in the multi-institutional @neurIST project. In the framework of this project the authors propose a strategy of federating data sources in the clinical institutions for use in research and in advancing clinical practice based on a real-life example. The authors also list security vulnerabilities, including the possibility of cracking the proposed pseudonym generation mechanism, dependence on a trusted third party, and the possibility of establishing indirect identification. However, they do not provide any solutions to these problems. Moreover, this approach only allows using data in the framework of a particular research project.

SciPort is a web-based collaborative biomedical data sharing platform that has been proposed by Wang et al. [27] to support data sharing across distributed organizations. SciPort uses a central server based data sharing architecture and provides collaborative distributed schema management across distributed sites. Our solution is close to the approach for sharing the data proposed in [27]; however, there are following important differences. Negotiation phase of our solution preceding the actual data exchange step allows the nodes to agree on the common schema for a particular database (instead of managing multiple schemas from different local servers). In our solution we minimize the use of centralized approach, by only employing it for P2P network organization (in contrast to sharing schemas through the central server as in [27]). Therefore, if the broker is temporally overcharged and is not available the peers can continue the data aggregation process within P2P networks that have been already organized. Finally, the authors do not discuss the need for data pseudonymization or anonymization assuming similarly to [6] that only the members of research consortia can access the data [27].

Several studies have shown that patients are concerned about their privacy, in particular in the case of medical data sharing: 62% of individuals worry that their electronic health records (EHR) will not remain confidential (Health Confidence Survey 2008, Employee Benefit Research Institute); 35% expressed privacy concerns regarding the publishing of their data to the database of Genotypes and Phenotypes (dbGaP) [28]. Therefore, it is unlikely that patients will be willing to share very detailed data as this can violate their privacy.

Need for anonymization and sharing individual patient data have been extensively discussed by the research community [4, 13, 29–31]. Several models in the area of distributed privacy-preserving data publishing have already been proposed (i.e., pseudonymization [6, 7], secure multiparty computations [8], microaggregation [32], and cloning [10]). However, those models significantly affect the quality and, therefore, the utility of data, since they do not take into account data availability, content, structure, and representation. The authors in [31] discuss the trade-off between privacy and utility of the data and the risks of breaking anonymity of the data. They state that the risk assessment has to be made for every single situation of data collection. We put this in place by allowing the peers to choose and negotiate the privacy parameters separately for every database.

An approach for continuous privacy-preserving publishing of data stream is presented in [33]. The authors use R-trees and allow the publication of data into the research database only after performing microaggregation locally. Similar to another approach based on two-phase microaggregation proposed in [9] the authors do not present any algorithm that allows the sources of data (medical institutions) to negotiate and to find an agreement on the characteristics of the research database (including anonymity parameters).

Release of only statistical data or providing only possibility to perform aggregation queries over the data as it is proposed in [34, 35] can guarantee the patients privacy. However, this may be not suitable for many types of medical research. For instance, Bellika et al. presented an agent-based distributed system for privacy-preserving statistical query and processing of EHRs in [34]. The role of the system in the proposed approach is to perform initialization and coordination of the distributed computation components among the sites participating in the computations. The advantage of the approach is that information transformation between information models for clinical use and statistical processing can be avoided. However, this framework cannot be used in the cases when the researchers need to access raw, not preprocessed data, for example, when having just a result of a query is not sufficient. Moreover, this approach does not take into account the possibility that the data about the same patient can be distributed between different sources (peers). Also, in the case of statistical query the peers are required to be always online and available to perform computations. In our system it is not mandatory. The node could potentially join the process of data aggregation if it is able to provide the data according to the conditions established during the negotiation phase.

Urovi et al. in [36, 37] proposed a secure mechanism for EHR exchange over a P2P agent-based coordination framework. In this approach the encrypted heterogeneous data are exposed over a P2P network. The authors provide the algorithms for searching and for publishing the EHRs in the untrusted P2P network without compromising the privacy, the integrity, and the authenticity of the shared data. Urovi et al. covered data aggregation from the perspective of finding the records of a patient. However, our focus is to create RSDB with the data about different patients. We extend the work of Urovi et al. by providing a way to collect the data about different patients from multiple sources and anonymize the patient's identity so that, even if records are shared in RSDB, the patients' privacy is preserved. The dynamic creation of RSDB was out of the scope in Urovi et al. In addition, we define a negotiation process for which these data can be aggregated dynamically. Nonetheless, the work of Urovi et al. shares some of similarities to our own, notably the use of TuCSoN coordination model [14] for agents negotiation phase.

MOSAIC [38] is a protocol for clinical data exchange with multilateral agreement. This system had two elements in common with our work: use of agents and a lookup system for peers to exchange data with. MOSAIC was designed to build research databases for private use, and thus, the data privacy is not taken into account in the design of the protocol. Moreover, it is also considered that the different institutions would optionally require more medical data in exchange as queries were made to them. As a result, contributor agents could optionally set a number of medical cases of a certain kind as a requisite, and other (petitioner) agents would have to resolve the requisites imposed by the contributor agents. This problem was solved through the use of multilateral agreement between agents. This is different from our work since we assume that medical institutions are willing to share the data for research purposes on a volunteer basis, knowing that secondary use of medical data can significantly enhance healthcare experiences for individuals [6] without looking for a certain profit but aiming at patient care improvement in general. Finally, this system is not capable of building a shared, anonymized research database.

## 5. Conclusions

We developed and implemented a multiagent system for dynamic aggregation of medical data for research purposes. The system allows facilitating and accelerating the process of data aggregation and building a research database with the possibility of updating it dynamically while preserving the patients' privacy. The data aggregation mechanism can be adapted based on the research study requirement on the fly. Negotiation between agents and data exchange have been evaluated using patients data collected in the neonatal intensive care unit over 5 years within the frame of a routine TDM program [3].

Apart from the “mutual gain,” creating the datasets that can be found and reused with respect to the requirements of a study, evaluation results demonstrate that the more time the system is in use the closer it is to a “fair” state, that is, when the difference between the number of records provided and the number of records obtained by an agent converges to zero. We believe that using our MAS will not differ significantly from the user point of view compared to using a single database. However, the advantage of using the system proposed is that it will offer access to more data, in a shorter period of time and in a privacy-preserving way. Integrating the system in a hospital environment for therapeutic drug monitoring is one of the next steps of our future work.

In the future work we will continue with evaluation of the generalization module that allows dynamic updates of the research database without violation of patients' privacy including quantification of data privacy and data losses. Currently we are developing an algorithm to improve the utility of the data when the database grows without violating the patients' privacy. We also plan to develop reasoning mechanisms to specify when the use of anonymization is mandatory and how to choose the parameters for anonymization.

## Figures and Tables

**Table 1:** Functionality and characteristics of virtual machines.

Name	Functionality	Guest OS	CPU	RAM	Disk
VM Router (router.virtualbox)	Routing traffic from the virtualization environment to the Internet, hosting a DHCP server and DNS server	FreeBSD 10.1 x86	1 core	512 GB	8 GB
Orion Context Broker Instance (orion.virtualbox)	Hosting an instance of the Orion Context Broker	CentOS 6 amd64/RHEL 6 amd64	2 cores	4 GB	20 GB
TuCSoN Node (tucsonX.virtualbox)	Representing a node in the network (also requires JRE 8 to run Java code)	Debian 8 amd64	2 cores	1 GB	8 GB
Database (mysql.virtualbox)	Acting as a MySQL server as a storage backend for medical data	FreeBSD 10.1 amd64	2 cores	2 GB	20 GB

**Table 2:** Evaluation of performance and scalability.

Number of agents, *n*	1	3	5	10
Time of a system run, *t* ^run^(*n*), sec	1.3	21.6	25.6	46.1
Time of local data collection, *t* ^loc^, months	60	—	—	—
Time of distributed data collection, *t* ^dist^(*n*), months	—	20	12	6
